# Modern Plastic Surgical Practice: Technical Competence Alone Is Not Enough

**DOI:** 10.29252/wjps.9.2.119

**Published:** 2020-05

**Authors:** Ankur Khajuria

**Affiliations:** 1 Department of Surgery and Cancer, Imperial College London, UK;; 2 Kellogg College, University of Oxford, UK

**Keywords:** Plastic surgery, Technical skills, Non-technical skills, Mentorship, Leadership

## Abstract

Annually, an estimated 234 million major surgical operations occur worldwide, with concomitant seven million complications and one million deaths. It is now well established that technical competence is necessary, but not sufficient for modern surgical practice and outcomes. Breakdown in non-technical skills has been attributed as a key root cause for near misses and patient harm in the operating room. This article discusses the multi-faceted skills-set that is necessary for the modern surgeon to succeed and for optimal patient outcomes. This includes technical skills, non-technical skills, with a focus on key CanMEDS framework domains, including leadership, communication, evidence-based surgery and mentorship.

## INTRODUCTION

Annually, an estimated 234 million major surgical operations occur worldwide, with concomitant seven million complications and one million deaths.^1^^,^^2^ It is now well established that technical competence is necessary, but not sufficient for modern surgical practice and outcomes.^3^^-^^5^ Breakdown in non-technical skills has been attributed as a key root cause for near misses and patient harm in the operating room. The complex inter-play between individual clinical skills, team factors and the clinical environment influences patient outcomes, as postulated by the systems approach.^5^

The CanMEDS clover, developed by Royal College of Physicians and Surgeons of Canada, is an educational framework that describes the key aptitudes underpinning the surgical expert.^6^ Both the profession and the general public expect to see these attributes in surgeons and these are widely accepted to lead to optimal healthcare and outcomes.^6^ This article will explore these aptitudes, and will discus both technical and non-technical skills. The non-technical skills section will begin with an overview followed by detailed discussion focusing on leadership (leader domain) and communication (communicator domain). Finally, evidence based surgery and mentorship (scholar domain) will be discussed as other key determinants of patient outcomes in surgery. 

## TECHNICAL SKILLS

Technical skills are an obvious prerequisite for good patient outcome.^7^ Indeed, Mahmoudi and colleagues showed that the risk of postoperative complications after free flap surgery was lower in high-volume and more experienced surgeons.^8^ The well-documented volume-outcome ratio provided further, emphasises the importance of technical skills with evidences supporting lower risk of operative death for patients treated at a high volume center.^9^

The lower mortality rate may, however, in part be explained by superior multidisciplinary care at the high volume centere and patient selection.^10^ The volume-outcome ratio has formed the basis of centralisation of surgical services for a variety of procedures.^11^ Technical skills learning has typically been delivered through an apprenticeship approach, whereby surgeons enhanced their skills through repeated practice on patients.^12^

The introduction of minimally invasive surgery was hailed as the “most dramatic change in surgery since the introduction of anaesthesia.”^13^ It has now led to several procedures being performed exclusively by the laparoscopic approach, such as cholecystectomy. However, this is only when the early part of the learning curve had been taken into consideration. Early complications sparked doubts regarding the procedure’s safety and this led to reconsideration of the training strategy.^14^

Skills courses were introduced for teaching basic psychomotor skills. As the number of surgeons performing the procedure increased, novice surgeons were then able to assist and learn. In Europe, the introduction of the European Working Time Directive (EWTD) with reduced training time,^15^ combined with an unprecedented need for patient safety due to high profile cases^3^ meant that alternative training strategies had to be sought. The operating room was clearly not a safe environment for such experiential learning to occur.^16^

Simulation provides a viable and valid alternative for technical skill acquisition in a controlled, safe environment with no harm to the patient, especially at the early part of the learning curve. It allows proficiency based curricula to be delivered, enabling structured training in the form of knowledge base, task deconstruction, laboratory environment training and skills transfer, with valid and reliable measures of assessment.^17^^,^^18^

Indeed, simulation has been shown to shorten the learning curve and lead to a faster rate of technical skill acquisition. Moreover, simulation and structured curricula allow trainees to exercise deliberate practice, which has been shown to improve technical skills.^19^^,^^20^ Furthermore, there is evidence to support skills transfer leading to improved performance in the operating room.^21^ However, technical skills are not sufficient for optimal performance and patient safety. 

## NON-TECHNICAL SKILLS

Non-technical skills can be divided into interpersonal (team-work, communication), cognitive (situational awareness and decision making) and personal resource skills (e.g. stress and fatigue management).^22^ Breakdown in these so-called ‘soft-skills’ remains the key root cause of near misses and patient harm in the operating room.^5^ They have also been shown to impact technical skills.^23^ Gawande and colleagues identified communication breakdown and fatigue/excessive workload accounting for 43% and 33% of incidents respectively at three teaching hospitals in USA.^4^

Such communication failures may occur across the entire continuum of a patient’s care.^24^ Indeed, employment of team training programs has been associated with reduction in surgical mortality.^25^ Moreover, several intra-operative stressors occur in the operating room that can impair a surgeon’s performance and compromise patient safety.^26^ Stress has been shown to impair psychomotor performance on a surgical virtual reality simulator.^27^

Sevdalis and colleagues demonstrated that case irrelevant communications (CICs) occur in the operating room and can interfere with sensitive work, especially during critical time points/steps of the procedure.^26^ Furthermore, the group correlated interference levels with the frequency of the operating room door opening.^28^ Noise in the theatre, which can reach 85 decibel, can lead to deterioration in the ability to communicate, increase stress levels and affect complex motor skills.^29^

Additionally, teamwork is a critical non-technical skill that influences outcomes. Mazzocco and colleagues demonstrated that when teams demonstrated infrequent team behaviours, there was increased likelihood of death or major complications.^30^ This has serious implications in the operating room, especially during crisis situations where teams may be more prone to error due to poor cohesiveness and communication.^31^ The next section will focus on key non-technical determinants affecting patient outcomes in surgery. These include leadership and communication. 

## LEADERSHIP

Leadership is a fundamental non-technical skill for a surgeon.^32^^-^^34^ It also directly influences other non-technical skills including teamwork, with poor leadership culminating in suboptimal teamwork and compromised patient safety.^35^ Moreover, surgeons are expected to** l**ead operative teams, execute multidisciplinary patient care, and engage in quality improvement. The rate at which improvements in performance are made is directly proportional to the quality of clinical leadership.^36^

Some surgeons also assume formal leadership positions at large institutions and healthcare organizations.^37^ Leadership has also been shown to impact technical skills.^23^ Expert surgeons believe that leadership is a critical non-technical skill that influences safety and efficiency in the operating room.^38^ Its inclusion in the non-technical skills assessment rating scales, include the revised NOTECHS, further exemplifies its importance.^39^

Effective leadership establishes a clear vision that is shared with individuals in an organization.^40^ This is particularly pertinent for quality improvement initiatives. The optimal conditions are created for individual and organizational success and to achieve significant goals.^41^ Pendleton’s primary colours of leadership model has three domains including (i) Strategic domain, which relies on intelligence, identifying problems and creating a vision/direction for team; (ii) Operational domain which relies on determination, executing the tasks, achieving results and (iii) Interpersonal domain, which relies on forming and sustaining relationships.^41^

A leader is responsible for setting a strategic direction, with clearly defined vision, values and analysis of contextual issues in an organisation.^42^ Quality improvement has been widely accepted as a means to enhance healthcare delivery.^43^ However, outside of trial settings, it has been difficult to entirely replicate the results.^44^ Key barriers include clinical staff engagement and the context within which the intervention takes place. These initiatives, if not aligned with strategic priorities, have limited longevity or impact.^45^

Alignment links the strategic domain to the interpersonal domain, relying on advocacy/negotiating skills and building and maintaining relationships with all key stakeholders to influence change and shape organisational culture.^46^ This represents Burns’ ‘transformational leadership’, distinguishing from the less potent ‘transactional leadership.’^47^

Transformational leadership stresses the importance of the relationship between the leader and the follower. It identifies the goals/needs and exploits the intrinsic motivators of both the leader and the follower towards a shared purpose.^48^ This facilitates effective team-working and is in contrast to the transactional model with the adage “You scratch my back, I scratch yours”, where there is no enduring relationship and the relationship is likely to disintegrate when individual parties no longer perceive that the relationship is likely to further their own interests.^48^

This links with the self-determination theory (SDT), distinguishing between intrinsic and extrinsic motivators.^49^ Intrinsic motivators facilitate one’s engagement in behaviour for its own sake, as it is personally rewarding. This contrasts extrinsic motivators, where behaviour is motivated by external rewards or to avoid punishment.^50^ SDT postulates three psychological needs that leaders can nurture in their followers, enhancing self-motivation and engagement. 

These include autonomy (individuals obtaining increased decision making authority to execute their primary work tasks), competence (aim of mastering one’s environment and outcome) and relatedness (need of close, affectionate relationships with others).^51^ To promote change, three key conditions as proposed by Ballard should be exploited.^52^ (i) Awareness of the nature of the problem to address, (ii) Alignment of objectives to the organisation’s strategic priorities, and (iii) agency (ability to change individual and organisational mind-set, through key stakeholder buy-in).^52^

However, of concern, is the finding that junior surgeons underrate importance of leadership as one of the CanMEDS roles and sense that current training does not ensure competence.^53^ Moreover, prior research has shown that surgeons are fairly accurate at self-assessing their technical skills, but lack the insight for non-technical skills self-assessment.^54^

It is paramount that training program directors ensure that importance of leadership is made explicit for trainees, with adequate training and assessment, through simulation as a possible avenue and utilizing validated assessment tools. The faculty/assessors themselves would require training to ensure assessment is reliable and valid.^55^ This should start from undergraduate through to post-graduate training to deliver high quality surgeons who have the abilities to lead multidisciplinary clinical and academic teams, and orchestrate change. 

## COMMUNICATION

In Europe, the EWTD has substantially altered working patterns, continuity of care and service delivery.^15^ With these changes and need for patient safety,^3^ high quality information transfer and communication (ITC) within and between healthcare teams is paramount for safe and effective patient care.^56^ ITC failures are ubiquitous in surgery, occurring across the entire continuum of a patient’s care and can lead to care provision errors and patient harm.^24^

Clinical handover is defined as transference of professional accountability and responsibility of a patient’s care to another professional.^56^ Effective communication is a prerequisite during this process to maintain continuity of care, and prevent errors, adverse events and patient harm.^57^ A number of checklists have been shown to improve quality and completeness of surgical handover.^58^ However, the findings must be interpreted with caution as majority of the studies lack randomization, blinding of outcome assessors and with no adjustment for confounding factors. Moreover, there is no investigation on the impact on patient outcomes following their implementation. On the contrary, when used sub-optimally or without stakeholder buy-in, checklists can have a deleterious impact on the surgical team functionality.^59^

Furthermore, Ghafferi and colleagues demonstrated a 2.5 fold difference in mortality in surgical patients between hospitals with comparable post-operative complication rates, but significant differences in failure to rescue (FTR) rates (i.e. mortality amongst patients with serious complications).^60^ Hence, whilst complications in the post-operative period may occur, quality of care (FTR being a marker of quality) and appropriate escalation, can determine patient outcomes.^61^

Here the advent of ward-based simulation may provide an avenue to train, enhance and assess multidisciplinary team communication.^62^ Validated, reliable and feasible tools, such as the quality of information transfer (QUIT) tool, can be utilized to assess quality of information transfer during escalation of care.^61^ This may form part of the next frontier in simulation, i.e. full-hospital simulation across the entire patient pathway.^63^

This may include a simulated patient admitted to the emergency department, reviewed by the surgical resident with a decision made to proceed to the simulated operating theatre with a computer-based mannequin simulator. This can be followed by a post-operative complication in a simulated ward environment that requires prompt recognition and escalation of care. It is paramount that more robust validated assessment tools are developed and that trainers themselves are adequately trained on how to assess. 

Finally, debriefing is a crucial component of Kolb’s learning theory and Schon’s reflective practice.^64^ Debriefing in surgery can decrease adverse events, enhance technical performance and facilitate deeper learning.^65^ It is also widely regarded as the most important component of simulation.^64^ However, there may be wide variation in practice. Guidelines on what constitutes an effective debrief are needed, to establish best practice and provide feedback to trainers.^66^

A tri-continental qualitative study established core components of an effective debrief.^66^ Validated, feasible and reliable tools such as the Objective Structured Assessment of Debriefing (OSAD) and feedback model, TeamGAINS have been designed to quantify debriefing quality.^67^ Utilization of these tools may facilitate a degree of standardization and quantification of the debriefing process to optimize surgical learning.

## EVIDENCE-BASED SURGERY

Clinical performance declines over time.^68^ Surgeons must demonstrate lifelong learning, contribute to scholarship and evaluate evidence.^6^ Evidence-based surgery is the amalgamation of the best available evidence with clinical expertise, patient and societal values.^69^ This facilitates rationale for decision-making and allows patients to make informed decisions. Surgeons must possess critical appraisal skills to evaluate literature for its validity, reliability and generalizability. 

However, hampering this process is the lack of high quality evidence in surgery, in particular Randomized Controlled Trials (RCTs).^70^ Poor reporting of surgical RCTs is also problematic and ubiquitous in surgery.^71^ Issues related to blinding, inconsistent care provider expertise and centers’ volume pose challenges both in conducting and reporting surgical RCTs, making it even more prudent that surgeons have the necessary skills to appraise such methodologies to inform their clinical practice. 

The scholar domain has been described as a ‘neglected competency in tomorrow’s doctors.’^72^ A survey of 515 medical students found that only 49% had understanding on how to appraise a paper and 22% had been trained on how to write a research paper.^73^ Reinders and colleagues showed that undergraduates who engaged in research produced four times as many publications compared to their colleagues and were more likely to pursue academia.^74^

However, barriers to undertaking undergraduate research, in particular intercalated degrees, exist including cost and prolonged training time. For those universities not offering intercalated degrees inclusive of the undergraduate program, alternative measures should be in place, including journal clubs or extra-curricular evidence-based medicine (EBM) workshops. EBM teaching may also be best delivered in clinics/bedside/ward rounds, where learners can apply EBM for real decision making.^75^ Critical appraisal skills should be nurtured early in medical school to cultivate understanding of responsible research and to develop future surgeons who have the ability to appraise literature and exercise evidence-informed decision making.^76^

## MENTORSHIP

The Editor of Plastic and Reconstructive Surgery exclaimed, “mentoring is one of the least expensive and most powerful ways to change the world.”^77^ A mentor helps to nurture technical and non-technical skills, professional values and attitudes. There is evidence to suggest that trainees with mentors demonstrate greater productivity, experience lower rates of burnout and have improved personal satisfaction.^78^ The importance of mentoring is supported by both the American College of Surgeons and the RCSEng.^79^

Previously, as per the Halstedian apprenticeship model, one mentor-mentee relationship would last throughout the mentee’s career. However, it is challenging to establish such relationships in modern practice. A national cross-sectional study of 565 UK surgical trainees revealed that 51.3% lacked a mentor.^80^ The rotational nature of training means trainees may rotate between different hospitals every 6-12 months, placing greater demands to establish long-term relationships, across different trusts and organizations. 

Moreover, there may be no financial/other incentives for surgeons to engage in mentorship. Lack of a well-qualified mentor has also been cited as a hindrance. A mentor should be interested in the process and be willing to establish a relationship by investing time, providing guidance and constructive feedback.^81^ Other factors such as ethnicity, culture, religion and gender may also hinder mentoring. 

However, a mentee must establish clear goals with the mentor and be responsible for their own learning. The time and commitment of mentors should also be recognized and rewarded. Ultimately, this may empower surgeons to unlock their full potential, culminating in improved outcomes. Technical skills are necessary, but not sufficient for modern surgical practice. The article has explored multiple facets of the CanMEDS framework. These are fundamental for a surgeon to develop an effective, safe and evidence-based practice that is underpinned with integrity and professionalism. 

## CONFLICT OF INTEREST

The authors declare no conflict of interest.
